# Conditions for Feasibility of a Multicomponent Intervention to Reduce Social Isolation and Loneliness in Noninstitutionalized Older Adults

**DOI:** 10.3390/healthcare10061104

**Published:** 2022-06-14

**Authors:** Jose Hernández-Ascanio, Pedro Emilio Ventura-Puertos, Manuel Rich-Ruiz, Vanesa Cantón-Habas, Ana María Roldán-Villalobos, Luis Ángel Pérula-de Torres

**Affiliations:** 1 Maimonides Institute for Biomedical Research, Reina Sofía University Hospital, University of Cordoba, 14004 Cordoba, Spain; jhascanio@uco.es (J.H.-A.); pventura@uco.es (P.E.V.-P.); n92cahav@uco.es (V.C.-H.); aroldanvi@gmail.com (A.M.R.-V.); langel.perula.sspa@juntadeandalucia.es (L.Á.P.-d.T.); 2 CIBER on Frailty and Healthy Ageing, Instituto de Salud Carlos III, 28029 Madrid, Spain; 3 Family and Community Medicine Unit of the Córdoba-Guadalquivir Health District, 14004 Cordoba, Spain

**Keywords:** nursing, social isolation, loneliness, aged, primary health care, feasibility studies

## Abstract

Aims: To identify the factors conditioning the feasibility of an intervention to reduce social isolation and loneliness in noninstitutionalized older adults from the perspective of the intervention agents. Design: A Dimensional Grounded Theory study conducted from December 2019 to January 2020. Methods: Twelve participants were recruited from an experimental study developed in a health district of a southern Spanish city. Data were collected through focus group meetings, individual interviews, biograms, anecdote notebooks, and the field diaries of two participants not included in the other techniques. Transcripts were analyzed using thematic analysis. Findings: Findings were divided into three themes: (a) the elderly between the walls of loneliness, economic difficulties, losses, and the past; (b) intervention agents/volunteers between the walls of inexperience in the management of psychological/emotional processes, lack of moral authority, and difficulty in planning results adapted to the (elderly) person; and (c) intervention between the walls of (interest in) company and assistance at home, lack of involvement (“waiting for you to save them”), and withdrawal/“abandonment”. Conclusion: A profile of the specialized intervention agent, professionalized (or at least a mentored agent), with both technical and relational competencies; a clear understanding of the purposes of the intervention (empowerment, as opposed to having company or being helped with household chores) and the commitment to active participation by the elder; or adequate management of the completion of the intervention (flexibility, attachment management) are some of the main factors contributing to the feasibility of these approaches. Impact: The findings have potential implications in the field of primary healthcare because primary and community healthcare services can implement corrections to the proposed intervention and ensure its effectiveness under feasible conditions. The nurse is shown as the most appropriate profile to conduct this intervention, although more research is needed to analyze the feasibility of this type of intervention in the daily practice of community nurses.

## 1. Introduction

Over the past few decades, Western societies are experiencing major changes in demographic and social structure, such as the rapid increase in the number of people over 65 years [1] or the change in the cohabitation pattern, with a significant increase in people living alone [1]. When these phenomena converge, they often involve phenomena of social isolation and loneliness. Numerous studies indicate old age as the most likely stage of experiencing social isolation and loneliness [2,3,4,5].

The experience of social isolation and unwanted loneliness in noninstitutionalized older adults is directly related to contextual elements [2,3,4,5]. Among the most significant elements of this last period is the outbreak of the COVID-19 pandemic. The World Health Organization [6] has already indicated the strong impact that quarantine measures have had on the psychosocial dimension of the subjects, and more specifically on social isolation and loneliness, in which noninstitutionalized older adults have been a particularly vulnerable group [6].

Due to the restrictions to curb the COVID-19 pandemic, we face an increased risk of social isolation and loneliness, so it is considered timely and relevant to inquire about interventions that can address these issues. Individuals who were previously well integrated may now face isolation from those close to them without the possibility of direct contact. It is also possible that, due to social distancing, feelings of loneliness may arise in individuals who did not experience them before. 

The emergence and exacerbation of both social phenomena have led to the emergence of numerous intervention proposals designed to mitigate the negative consequences of these phenomena on the health of this population sector [7,8,9,10,11].

A significant higher interest in designing and validating intervention strategies in unwanted loneliness and social isolation of the elderly was found [9,10,11]. However, no attention is paid in the literature to some relevant issues, such as acceptability, sustainability, or usability of these strategies in socio-health care services, which are of absolute importance. Social isolation and unwanted loneliness in older adults are major problems that require high levels of complexity to be addressed [5], and so far, the literature shows that, despite the interest in the subject, there are relatively few interventions that are institutionalized and standardized in the field of health services [7]. It is necessary to go deeper into the factors that condition or hinder this incorporation in order to be able to carry out an adequate planning of this type of intervention.

From this perspective, there is a pressing need to know how it is “feasible” to carry out a program evaluation.

This feasibility study of the intervention requires two fundamental aspects to be considered: its “usability” (here, the acceptance of the intervention by the different actors involved to be implemented conventionally [12]), and its “sustainability” (here, the extent to which the intervention can be implemented continuously over time, maintaining its effectiveness features, with particular regard to the necessary resources and the context in which it is developed [12]).

In this context, the main objective of the present work was to identify which factors act as conditioning factors in the viability of an intervention specifically designed to reduce social isolation and loneliness in noninstitutionalized older adults, using the perspective of the intervention agents as a reference. The choice of intervention agents is justified by the fact that the main interest is to investigate the factors that affect the intervention delivery system.

The question that guided the present work was what are the factors associated with the agents, the participants, and the dynamics of the intervention that condition feasibility.

## 2. Background

The meta-analysis conducted by Masi et al. [13] divides intervention strategies into four types: those increasing social skills, those designed to strengthen social support, those increasing chances for social interaction, and those for socio-cognitive training. However, another more recent review [9] identifies more than 44 experiences that could be clustered into nine different types of intervention, classified according to their guidelines for addressing social isolation and loneliness in older adults: (a) personal contact; (b) activity and discussion groups; (c) contact with animals; (d) skills course; (e) support group; (f) care model; (g) reminiscence; (h) varied non-specific, and (i) public dissemination.

As the literature [13] shows, multiple strategies have been followed to develop intervention projects in the field of unwanted social isolation and loneliness in older adults. However, the available evidence [7,8,9,10,11,12,13] highlights several associated features with increased effectiveness. In this sense, the main features are (a) interventions with theoretical underpinning and methodological soundness vs. those without; (b) multi-methodological interventions vs. those with a single methodology; (c) interventions based on participatory designs vs. those based on expert knowledge only; (d) interventions with process evaluation vs. those with only an outcome evaluation; (e) interventions with specific training and prior training of intervention agents vs. those based on generalist knowledge; (f) interventions with an educational component, group-based, and a particular target population vs. those that do not involve behavioral changes, of an individual or generalist nature; (g) interventions that seek synergies with community resources vs. those that make use of hyper-specialized resources; and (h) interventions based on personalized itineraries vs. those based on standardized interventions.

The previous literature has shown that the content and modality of these interventions condition difficulties in their implementation, highlighting some problems related to the recipients of the intervention, such as the lack of participation or involvement of the elderly [14,15]. Others are related to the agents or facilitators of the intervention. In this regard, the literature points to difficulties related to the inadequate selection or training of the intervention agents, the lack of support during the implementation of the program, or those resulting from the emotional work of the facilitators (who may experience overwhelming responsibility) [16,17].

Given this background, the present study analyzes the feasibility conditions for this kind of intervention, specifically a modification in the Carelink intervention [18]. This intervention is of a multicomponent nature, combining actions aimed at active search, re-engagement, and the direct intervention on the subject. It should be noted that this is an individualized intervention, developed over 4 months by means of a home visit, where the intervention agents were students of health sciences degrees, volunteers from social organizations linked to the issue, and health professionals. This intervention also proposes a process of progressive empowerment of the elderly in such a way that they actively participate in decision making and in the planning of an important part of the contents and format of the intervention. 

Among the most noteworthy contents of the intervention is the consideration of motivational, educational, skills training, and physical reactivation aspects. The efficacy of all these aspects is supported by the available evidence [7,8,9].

It is important to note that our study provides forms of evidence that are not found in the effectiveness reviews performed to date for this type of intervention (suitability, acceptability, usability, etc., of interventions) [10,11,12] which were focused only on quantitative studies. Moreover, these aspects are central elements of our analysis proposal.

## 3. The Study

### 3.1. Aims

To identify the factors that have conditioned the feasibility of an intervention to reduce the social isolation and loneliness in noninstitutionalized older people from the perspective of intervention agents.

This study complements the results of a multicenter, parallel two-group, cluster-randomized controlled clinical trial conducted between April 2018 and December 2019. The objective of the clinical trial was to validate the effectiveness of a multicomponent intervention, developed in primary health care services, in the field of social isolation and unwanted loneliness in noninstitutionalized older adults. 

More detailed information on the clinical study research protocol can be found in the published version of the study [19], recorded on ClinicalTrials.org (NCT03345862).

**Figure 1:**
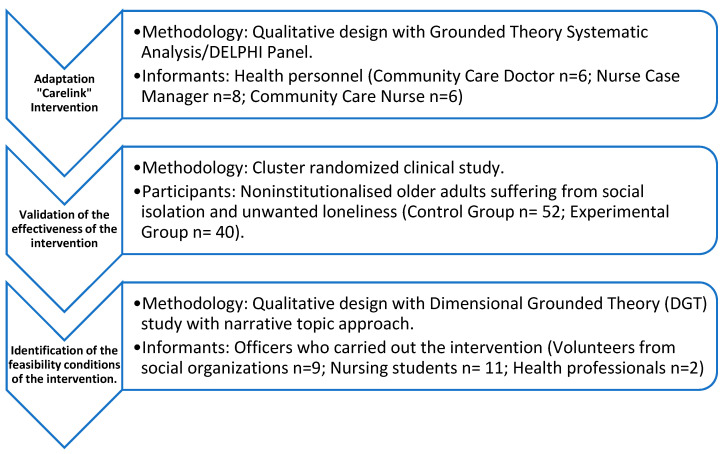
Flowchart on the overall configuration of the global project.

The following is a flow chart showing the different studies that made up the overall research project (Figure 1).

### 3.2. Design

A Dimensional Grounded Theory (DGT) study with narrative topic approach. The Dimensional Grounded Theory (DGT) [20] is a methodological proposal that implies a revision of classical Grounded Theory, whose main particularity is that it, as opposed to the pretension of establishing a substantive or formal theory, pays more attention to explaining the process of selection and organization of the dimensions that are used in the definition of situations or the construction of meanings. The choice of this methodological proposal is determined by the fact that, by placing greater emphasis on the formal dimension of the categories than on their final contents, it can serve to establish similar evaluation processes in the context of other interventions in the future, allowing for the systematization and standardization of the results.

Consolidated criteria for reporting qualitative research were followed for the design.

### 3.3. Sample/Participants

The selection of informants was carried out using a structural design concerning the study population (agents who participated in implementing a multicomponent intervention to reduce social isolation and unwanted loneliness in noninstitutionalized older adults). 

An intentional sampling (based on a careful and detailed assessment of the possibilities of subjects to provide in-depth and detailed information on the objective of the research for those agents who completed the intervention in the experimental study). Consequently, those participants who had shown higher levels of participation and better communication skills during the project’s development, in the opinion of the project coordinating team, were taken into account. As Morse [21] indicates, the collaborative and proactive attitude of the informants is a facilitating element in the in-depth access to information, which is why this element was established as a complementary criterion for the selection of informants. 

A critical case sampling (for intervention agents who dropped the experimental study entered as informants, although quantitatively they were a marginal number). 

**Figure 2:**
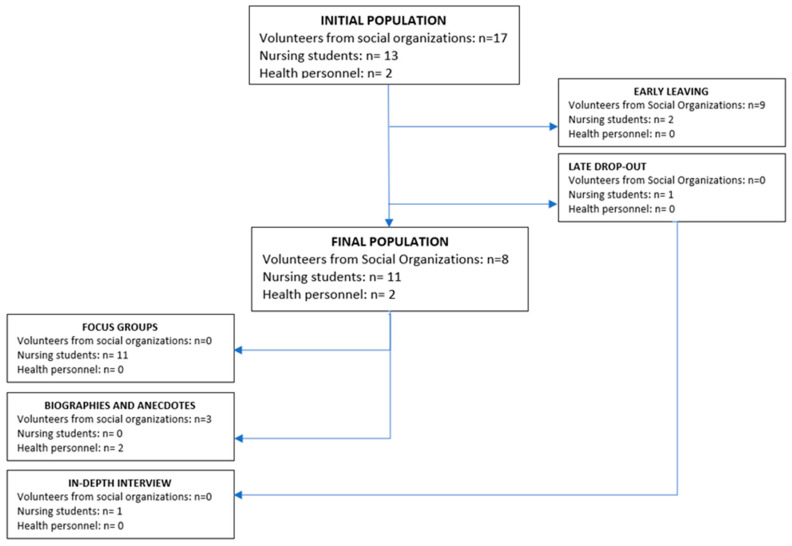
Study sample.

The following participated in the project as intervention agents: 17 volunteers from social organizations (8 of whom dropped out), 13 nursing students (2 of whom dropped out), and 2 health professionals (Figure 2). In the case of the volunteers belonging to social organizations who dropped out, they did so early (after having completed the initial training and having had the first encounter with the assigned older adult in the study) and for reasons unrelated to the intervention. 

However, the number of study cases (groups, interviews, or documentary resources) was not defined in advance. The collection of information continued until the saturation of speech occurred. To confirm the achievement of the principles of theoretical and thematic saturation, the analytical recommendations of this critical version of Grounded Theory [22] were applied, consisting of contrasting the application of the method of constant comparisons to a theoretical sampling with the theoretical sensitivity of the researchers and subjecting this contrast to a triangulation of researchers. After the application of these criteria, the final number of informants was 12.

The following table (Table 1) summarizes the main definitive characteristics of the informants.

Participants were recruited from among subjects who participated as RCT intervention agents. Members of the research team carried out recruitment through direct contact with the interested individuals based on a careful and detailed assessment of the possibilities of subjects to provide in-depth and detailed information. 

### 3.4. Intervention

The tested intervention is a modification of the Carelink intervention [18]. This modification was introduced due to the difficulties faced by health professionals for the feasibility of the intervention in our primary care setting. The changes affected the number, duration, and frequency of the sessions. The content of the activities was maintained. The modified intervention consisted of six visits to the home of the elderly and five telephone calls. The visits lasted 30 min each over 16 weeks. The telephone calls lasted approximately 20 min each. The face-to-face and telephone calls were interspersed according to the characteristics of each person. The first visit, aimed at setting objectives and building a trust relationship for future visits, lasted one hour. The pre-established activities were aimed at social interaction and contact (conversations and discussions about current news), to increase feelings of competence and personal control (evocation and reminiscence, identification of causes, planning of activities, and training in coping skills, among others), and to participate in social activities (information on social health resources).

### 3.5. Data Collection

Three different types of techniques were used for data collection: (a) focus groups, (b) semi-structured interviews, and (c) biogram analysis, anecdote notebooks, and field diaries.

1. The application of these techniques was of a segmented nature so that:
2. Two focus groups were carried out, composed of nine subjects. The focus group was performed with agents who had an average of 2–4 intervention cases assigned and successfully completed the project. Two discussion groups (each lasting approximately 2 h and with the participation of 9 subjects) were carried out, jointly conducted by one member (MSc) of the research team and an independent observer.
3. The in-depth interview was reserved for one of the participants who abandoned the project early. The interview was conducted by a member (MSc) of the research team.
4. The analysis of biograms, anecdote notebooks, and field diaries was performed on agents with more than 6 cases assigned. In this case, two informants participated in this technique.

The recruitment of informants according to the techniques applied was carried out following the recommendations of good practice in qualitative research proposed by Fernández de Sanmamed and Calderón [23].

For the development of the focus group and interview, a starting script was developed. The categories in this script were: (1) characterization of participants; (2) prior views and beliefs about social isolation/loneliness; (3) intervention process: pre-phase, implementation and evaluation of results; and (4) personal assessment of participation in the project: emotional and formative involvements. 

The initial categories used for the elaboration of the interview scripts and focus groups are the result of an emergent analysis of the biograms, anecdote notebooks, and field diaries that were carried out with the agents with more than 6 assigned cases.

Based on the analysis of these materials, the interview and focus group scripts were elaborated in order to triangulate the contents and allow for their complementation [23].

Data collection took place between May and June 2020.

### 3.6. Ethical Considerations

The study was performed following the ethical principles of the Helsinki Declaration [24] and has the permission of the Ethics Committee for the Province of Córdoba (Spain). 

Before the interview and focus groups, participants were informed about the study and provided their informed consent. At all times, the person’s anonymity was guaranteed. For this purpose, the responses have been anonymized using pseudonyms. The personal data obtained have been processed following the General Data Protection Regulation EU/2016/679, of 27 April 2016, and the provisions of the Organic Law 3/2018, of 5 December, on the Personal Data Protection and Digital Rights Guarantee.

### 3.7. Data Analysis

The method of constant comparisons [25] supported in the Weft-QDA tool has been used for data analysis. The analysis was divided into the following phases: (1) reading of materials and choosing topics of interest (codes), (2) discussion and agreement with the tutor of topics of interest (codes), (3) rereading of materials and coding (extraction of contents of interest classified by topic), (4) analysis by topic/codes, identifying regularities and differences (constant comparison), and (5) development of a conceptual map for the synthesis of findings, based on the information found in the interviews. In addition, analytical and theoretical memoranda were prepared to develop and define the categories. The textual analysis was complemented by the situational analysis of frameworks [26]. The analysis was performed by two of the researchers independently.

### 3.8. Rigour

To ensure the rigor of the findings, each resulting category was contrasted with the original transcripts. In addition, during the interviews, or immediately at the end of the interviews, summaries of the contributions were made or confirmation was required from the informants to verify the information. On the other hand, different ways of obtaining information have been used, researchers have been triangulated in the analysis, and a constant interaction and dialogue with the researchers’ theoretical–conceptual frameworks has been sought. Likewise, reflexivity (the constant analysis of the indisputable role that researchers have played in producing the findings) has always been present in the study.

Finally, it should be noted that a rich and deep description of the phenomenon studied has been made to guarantee transferability, that is, the possibility of generalizing the findings obtained to another context whose meaning is similar to that of the context studied.

## 4. Findings

The findings obtained from the participants’ discourses can be grouped around the following three themes: (a) the elderly between the walls of loneliness, economic difficulties, losses, and the past; (b) intervention agents/volunteers between the walls of inexperience in the management of psychological/emotional processes, lack of moral authority, and difficulty in planning results adapted to the (elderly) person; and (c) intervention between the walls of (interest in) company and assistance at home, lack of involvement (“waiting for you to save them”), and withdrawal/“abandonment”. 

**Figure 3:**
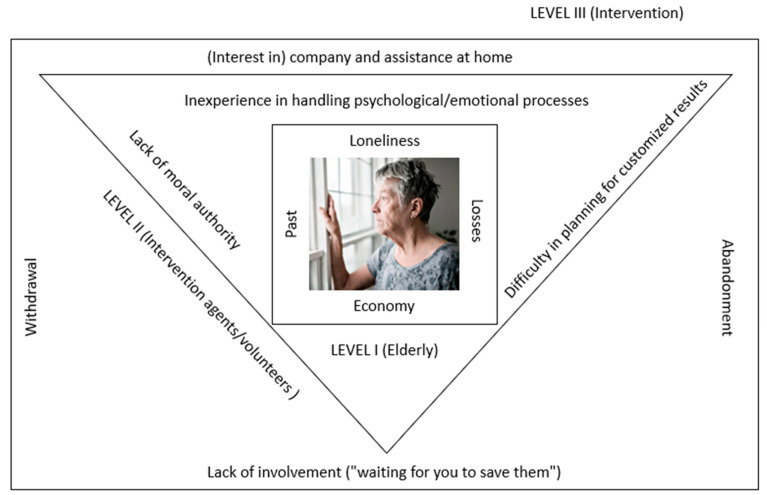
Factors conditioning the proposed intervention.

Figure 3 represents the relationships between these themes and their main categories, representing (ultimately) the factors that conditioned the feasibility of the proposed intervention.

### 4.1. The Elderly between the Walls of Loneliness, Economic Difficulties, Losses, and the Past

From the participants’ perspective, the first conditioning factors were related to the personal circumstances of the elderly being cared for. The fundamental ones were that the subjects were elderly living alone, with some limiting physical, psychological, or social condition, without social support networks, and with significant changes in the family structure or estrangement from close relatives (“*having no husband, no wife, no longer having that someone they used to live with at home*”, I2G1; “*and obviously having certain limitations, that they could not have a life beyond the four walls of their house*”, I8G1; “*children have their own life*”, I4G1; “*previously, there was a much closer relationship with his daughter, but as she now has her children, then he has to leave her space*”, I1E1).

Architectural barriers, such as the lack of elevators or the non-adaptation of the home, together with economic problems/belonging to a neighborhood of social transformation, also appear as essential conditioning factors (“*This man lived on a fifth-floor without an elevator, he had problems in the legs, could not walk*”, I8G1; “*In his house, he had to go downstairs and if he could not, then he would stay at his house and that’s all*”, I1E1; “*he was living in those flats, in that neighborhood, it was a bit more complicated for this him*”, I1E1).

The volunteers expected all these conditions. However, among these, a group of conditioning factors of great importance emerged: the unresolved emotional adaptation processes, such as the existence, in the past of the elderly, of a series of experiences that conditioned their present, and the recent experience of a bereavement situation (“*the case is that one of them felt very abandoned by her children and sisters. They have had several misencounters, and in recent years they stopped talking to each other. The woman was very proud and preferred to continue in the same way instead of recognizing that she had been wrong and taking the first step*”, C1; “*the man was there in full grief due to the death of his female partner*”, I5G1).

### 4.2. Intervention Agents/Volunteers between the Walls of Inexperience in the Management of Psychological/Emotional Processes, Lack of Moral Authority, and Difficulty in Planning Results Adapted to the (Elderly) Person

#### 4.2.1. Inexperience in the Management of Psychological/Emotional Processes

Linked to the last group of conditioning factors that have been pointed out (unresolved emotional adaptation processes), the intervention agents reported being overwhelmed by them, considering them unapproachable by volunteers (“*I find myself very small to help them in that sense*”, I6G1; “*you do not know how to deal with such murky issues*”, I6G1). They considered it to be outside their scope of competence (“*She needed a psychologist, I pressume, and we could not provide her that*”, I5G1).

Therefore, it is essential to consider/ensure the training/competence of intervention agents to address psychological/emotional processes, often unresolved in intervention subjects, as one of the central conditioners of feasibility linked to these agents.

#### 4.2.2. Lack of Moral Authority

In addition, the volunteers in our study believe that they lacked the moral authority that the elderly give to health professionals (“*I was encouraging her throughout the project to go out, and at the time her nurse told her that she had to walk because of her diabetes, she was going out every day*”, I4G1). The volunteers coincide with the vision that the elderly had of their role: they saw the volunteer as a grandchild, who came to give them company (“*I sincerely saw myself in the role of a granddaughter*”, I6G1), and not as a professional who came to ask them to make an effort on their part (“*They were for the accompaniment, but they did not want to do anything else*”, I2G1). The volunteers felt like strangers in the older adult’s home and even felt vulnerable (“*I find it hard to enter a house of a person I do not know. At the hospital, you’re in your area, and they come to you. Otherwise, it is you who gets into their environment, then you are completely vulnerable*”, I5G1). Thus, it is also necessary to consider (e.g., in the case of intervention carried out by volunteers) the adequate empowerment of the intervention agent as a new condition for its feasibility.

#### 4.2.3. Difficulty in Planning Outcomes Customized to the (Older) Person

With inexperience in handling unresolved emotional adaptation processes and the lack of moral authority, the volunteers/intervention agents pointed out the difficulty they encountered in planning results adapted to the person. In this sense, they considered the applied training carried out through individual cases as fundamental (“*I think the presence of tutorials is better, because... tutorials provide you tools applied to your case and do not limit you at the same time*”, I4G1). Despite the difficulties, the mentoring work discovered several standard lines: firstly, to facilitate the elderly to recognize their isolation situation and look for the causes of this fact (“*above all, that they admitted their situation, which they admitted it partly*”, I1G1). Once this has been achieved, define objectives appropriate to the person’s circumstances: improve telephone communication with the family/relatives (when there were difficulties in going out in the street) (“then I proposed the elder that instead of calling them once a week, calling them every day”, I6G1); going for walks (“*then, based on that, I thought it would be ideal to go out in the neighborhood because there are many bars, parks and so on in the neighborhood*”, I6G1); or (whenever possible) looking for collective activities offered by the community (“*I told the elder if he had thought about going to a workshop or something, to interact in the evenings with more people of his age*”, I5G1) for the elder to socialize with other elders in their community. The capacity of the intervention agents to plan/design outcomes following the personal and social capacities of the elderly thus becomes the third conditioning factor linked to the intervention agents.

### 4.3. Intervention between the Walls of (Interest in) Company and Assistance at Home, Lack of Involvement (“Waiting for You to Save Them”), and Withdrawal and “Abandonment”

One fundamental determinant of the intervention is its capacity to respond to the interest of the beneficiaries (the elderly). In this sense, informants emphasized that many older adults participated in the program because they believed that it consisted of keeping them company or helping them with activities of daily living (“*in some of the cases I found, on the first visit, the elder was somewhat confused about what we were actually going to do. His doctor had vaguely told him that a student was going to visit him and give him company and help him not to feel so lonely* “C1; “[…] *they were limited to just me going there to make conversation, and no much more*”, I3G1; “*The man was the caregiver of the woman who was practically dependent. Then the man had the idea that I was going to be with his wife. That I was only going for her, while he was resting a little bit, although he was there always*”, I3G1).

In this way, the visits became a more ample space than established (“*I was going to his home, and he spent hours and hours talking with me*”, I5G1). Nevertheless, the opposite was also the case (when they discovered that it was not an accompaniment program), with evasions/unavailability of the elders for the visits (“*many times they did not like it*”, I3G1; “*and I was told that he was on the “tuna” (listening to the university music group). I called him/her the next day and was doing something else. Then during the week occurred the same. He couldn’t go some days because he was attending a course with his wife*”, I5G1). Volunteers even believe frequent “abandonment of the intervention” was due to a lack of interest in this type of proposal (“*they had some expectations and thought it was one thing, they have found another*”, I5G1). Many of them “*were there due to the accompaniment but didn’t want to do anything else*”, (I2G1).

#### 4.3.1. “Waiting for You to Save Them”: Lack of Involvement

(“*They think: You send me this, give it to me; but they expect you to save them, when they should save themselves taking advantage of the resources*”, I4G1). Therefore, it is essential to know the interest of the older person in autonomy or capacity for self-government. The recognition in the older person of a proactive attitude and a desire to be responsible for themself is a necessary condition for the success of this intervention. (“*I believe that as an intervention that is based on cooperation, if it is true that the participants must have an attitude, a certain interest and above all maintain an attitude, as far as possible, a little proactive*”, I7G1).

In this sense, volunteers believe that it is essential to ensure that the person is aware of the need to actively participate in the program to guarantee the success of the intervention and that, if this is not the case, other different interventions should be considered (“*I think that if they had already arrived with the idea that…, look, this consists in that, step by step, you can go out to the street by yourself. Then I think that would have been a good incentive, but nothing*”, I3G1; “*Maybe if the elder recruited had got a clearer idea about the real expectations they could consider, what were the expected demands on them at the level of involvement and commitments, the results would have been different because they would have been much more motivated with the project and had shown less resistance*”, C1; “*It is something with much ambition, then, you have to be motivated for that, at the moment when you are not motivated, I do not know what other intervention, but not this one*”, I4G1).

#### 4.3.2. Withdrawal and “Abandonment”

Finally, the volunteers considered it essential to adapt the duration of the intervention to the needs of the individuals. If the objectives are not met in the last stipulated visit, it becomes necessary to consider extending the intervention (“*if you realize that a person has more difficulty, or has fewer resources, so extend it a little more*”, I5G1).

However, they consider that the duration (and, therefore, termination) of the intervention should be planned at the operational level, depending on the achievement of the objectives, and the emotional level. Informants say that the farewell was too open and that the last visit was not understood as the end of the intervention (“*I think it was very open and, well…, he thought we would meet again*”, I2G1). Thus, many volunteers shared a “sense of abandonment” at the end of the intervention program. Many felt that they “abandoned” the elder (“*it seems as if we were leaving them*”, I2G1; “*I have spent X days going to visit this lady, this lady has told me about her stuff and then I left. I didn’t feel well, truly*”, I5G1). In addition, they feared that the intervention would have a rebound effect, because many of the elderly never felt like working on their autonomy, but instead that they were in accompaniment (“*I think there may have been boomerang effect because of the simple fact that they did not have clear objectives*”, I4G1). Although the older adult’s interest undoubtedly influences this aspect in the accompaniment, this aspect should be considered, in any case, to ensure the non-attachment of the person to the intervention agent.

## 5. Discussion

First, the findings confirm the fundamental role of the conditioning factors associated with the characteristics of the older adult, mainly the condition of living alone and the feeling of loneliness. However, after learning the reality through experience, the informants in our study recognized that, although living alone may be associated with a greater likelihood of developing social isolation, the isolated older adult does not always live alone [27]. Therefore, it was understood that other situations are contributing to this isolation and that they have more influence, such as the loss of a spouse or the emancipation of children [28,29]. These findings are consistent with what Evans et al. [30] reported in their study; they argue that living alone and being isolated may be related, but the second situation is not a direct consequence of the first. The study insists that older people who live alone and are more isolated in terms of family network deficits often compensate for this lack with supportive relationships they establish outside the home [30], which to some extent is contrary to the initial perceptions of the informants.

Regarding the conditioning factors related to the agent of the intervention (volunteers, mainly nursing students), we found few references to interventions performed by students in the homes of isolated elders, but we found some interventions that have taken place in care homes. Annear et al. [31] analyzed the effectiveness of a student intervention in residential aged care facilities. The research concluded that the “youth” of volunteers was a positive factor contributing to achieving the objectives. Most elders living in these care homes mentioned that the students brought the “outside world” and vitality to the facilities [31]. The study conducted by Santini et al. [32] shows that intergenerational practices can be effectively integrated into interventions on the older population [32]. However, in the present study, the issue of youth appeared to act adversely by subtracting credibility and moral authority from the students who acted as intervention agents.

Conceivably, these differences are due precisely to the influence of the “intervention environment” identified by the informants, an interpretation that previous studies would support [33,34,35,36,37,38,39] that point to this variable as a significant differentiating one. In the prevention of social isolation, higher effectiveness of group activities performed in centers attended by community-dwelling elders has been previously demonstrated [9]. However, this is not always possible (in older adults who rarely attend a center voluntarily), and it is then that, as in our study, more customized interventions, with a home approach, are necessary to ensure that the older adult becomes engaged with the established network of social and health resources. Nevertheless, despite proving to be a more effective environment, the intervention environment stands out for having provoked a feeling of vulnerability in the intervention agents in our study. The informants described having felt vulnerable in the home of the elderly. This vulnerability does not appear in studies with students as intervention agents in elderly care homes [9]. No study explaining this perception was found.

On the other hand, informants highlighted the “emotional work” of this experience, which has, sometimes, been identified as an overload, a sense of frustration, and an “excess of responsibility.” These feelings have also been reported previously by Cotterell et al. [10], Franck et al. [39], and Gardiner et al. [11], who have shown that the emotional bonds created between the student and the user could become a negative emotional experience for the former when faced with contexts of frustration because they were not making progress in the proposed goals, because of the excessive demand and dependence of users and similar elements such as being fed up.

To prevent these effects, the informants point to the need for more comprehensive and more mentoring programs to improve the skills and competencies of the intervention agents. In this sense, the study of Dickens et al. [40] recommends the presence of service coordination figures as a strategy to minimize the negative effects of the complex nature of most known interventions on the subject. The study of these authors shows greater effectiveness when there is a case coordinator. Despite the existence of tutoring during fieldwork, our study has lacked the formal designation of this figure.

The self-perception of students’ lack of competence and authority and the influence they observe on the effectiveness of the intervention in our study is consistent with the information reported by Santos-Olmos [41], who points out a direct relationship between the effectiveness of the intervention and that it is conducted with highly trained and motivated professionals who are in line with the objectives of the intervention.

In this sense, the informants of our study consider it appropriate (and necessary) to assign the role performed by the volunteer intervention agents (students) to qualified health professionals (as already expressed in the study of Santos-Olmos, [41]). This suggestion is also pointed out by Sundström et al. [42], who recognize that the health professional has the right characteristics to alleviate the social isolation and loneliness of older people. In addition, Gardiner et al. [10] point to community nursing staff as the most resolvent professional profile, perhaps, as Sundström et al. [43] state, due to the increased ability of nurses to sustain a holistic approach.

Concerning the conditioning factors of the intervention, previous studies point out the importance of adequate preparation of the intervention. One of the greatest weaknesses identified by our findings has been the improper diligence of the superintendents/facilitators (health professionals) responsible for recruiting the participant elders. The informants believe that the elder’s clear understanding of the intervention purposes was not guaranteed, and no adequate assessment of the interest/acceptability of the intervention was performed. In this sense, the importance of adequate action by the facilitators is also pointed out by Santos-Olmos [41] as a factor directly related to the effectiveness of the interventions. Linked to the recruitment of intervention subjects, Santos-Olmos [41] reports on the important influence that the motivation of potential users will have on the effectiveness of the intervention, to the extent that the more motivated and involved in the intervention the subjects are, the results of the intervention are presented as more effective (an aspect that also appears as a weakness in our study).

Regarding the expectations of the elderly (to be accompanied) versus the desire of playing an active role in the intervention, our findings are consistent with previous studies [43,44,45] pointing to participation and co-responsibility as determining factors in the effectiveness of the intervention. The review performed by Gardiner et al. [11] notes that interventions involving active and productive participation of the elder are more useful in alleviating social isolation than those involving passive activities or having no specific goals [11].

The active participation of the elderly is also one of the leading indicators of the effectiveness and sustainability of the interventions identified by Santos-Olmos [41]. In her study, this author confirms through an empirical study discussed in systematic reviews [11] that when the elder shows they are feeling highly engaged with the intervention proposal, and this includes the mobilization of different capacities and skills of the elder, the findings are significantly better and more lasting than other interventions in which the elder acquires more passive roles.

Although the program aimed at the active participation of the elder, the informants point to a clear passivity of the elder. The reasons for this position could be that no one clearly explained the project’s objectives in the greatest way (“or who did not know/wanted to understand them”), that it was difficult to lead wrong expectations during the intervention (“if they only expected company”), or that, actually, the elderly did not play an active role in defining the objectives (a fundamental issue, according to Drentea et al., [46]), with most of the individual objectives being the product of the suggestion of the intervention agents rather than the users themselves.

Concerning the content of these objectives, although in some cases it was possible to improve communication with the family, that the elderly goes out or even participates in group activities, in no case was a group activity bringing into contact the isolated elders participating in the project, despite the demonstrated effectiveness of this type of activity [47].

Finally, our findings point to the requirement to tailor interventions to the specific needs of each elder and the importance of flexibility if these individual needs are to be met. This fact is pointed out by previous studies [11], which, in addition, point out the necessary flexibility when setting the schedule of intervention sessions [46], a measure that facilitates user adherence to the program.

Thus, adaptability, flexibility, and active participation of the elderly, among others, constitute potential factors that would improve the feasibility of the project [48].

### Limitations

The limitations of the study concern primarily the modification of the Carelink Program. The study does not examine the Carelink Program [16], but rather a modification made due to the difficulties encountered by health professionals in the feasibility of the intervention in primary healthcare in our environment. In addition, and because the modified intervention was also difficult to implement by health professionals, the intervention was finally performed by voluntary staff (nursing students and NGO members).

These changes have prevented identifying the main factors affecting the feasibility of intervention from public health services, as stated in the original research protocol [19]. This feasibility analysis was performed, as noted above, through a previous qualitative study.

In addition, it is necessary to recognize a possible bias resulting from the sample selection of informants, as access to critical cases such as agents who abandoned the intervention was limited (only one of the four who left the project early could be contacted).

It is also necessary to point out that, in the group of volunteers from social organizations, 8 of the 17 volunteers who started the intervention dropped out. However, their characteristics did not differ from those who remained, and the causes for dropping out were unrelated to the intervention implemented.

In addition, it is also possible to identify a possible bias of information derived from the use of biograms and anecdote notebooks: Although standardized data collection tools were provided and informants were advised of the way information collection would occur, the open nature of these tools made their standardized use difficult. However, to minimize biases resulting from the subjective completion of these forms, periodic revision of these materials was performed, which also helped to prepare the process of monitoring the intervention itself.

As well as those derived from the complementary study (on the effectiveness of the multicomponent intervention)—in which the participants of the present work tried to reduce social isolation and unwanted loneliness in noninstitutionalized older adults—we believe that the results of this study are particularly relevant in the current post-pandemic context of COVID-19. In this context, the elderly who were previously well integrated now have to face a scenario of more minor interactions with those close to them, which can generate feelings of loneliness in these people who once did not experience it.

## 6. Conclusions

The findings of this study have revealed significant elements that can act as conditioning factors for the feasibility of interventions in the field of social isolation and loneliness with noninstitutionalized older adults.

In the first place, we find conditioning factors associated with the characteristics of the older adult (loneliness, economic difficulties, losses, and the past, among others), but unresolved emotional adaptation processes are particularly complicated.

Secondly, there are conditioning factors associated with the competence of the intervention agents (volunteers/students), mainly inexperience in managing psychological/emotional processes, lack of moral authority, and difficulty in planning results adapted to the (older) person. These shortcomings demonstrate the need to possess (or at least train) psychosocial intervention skills and determine a profile of the specialized intervention agent (or at least mentored work). In addition to specialization, participants suggest the need to professionalize the intervention (with the authority of a given profession).

Third, the importance of ensuring that the intervention is in the best interest of the beneficiaries has been emphasized. Consequently, it is required that the elder has a clear understanding of the intervention’s purposes and that the practitioner has an adequate assessment of the interest/acceptability of the intervention. From the beginning of the intervention, the professional should explain the goal of empowerment (as opposed to keeping company or helping with household chores) and the commitment to the elder’s active participation. The need for informed participation is highlighted as a fundamental condition for viability. The lack of this condition means that the expected results are not achieved, and it also leads to frustration among the intervention agents because the initial expectations are not met.

Finally, another fundamental aspect related to the above is to mention explicitly (from the beginning of the intervention) and to remember (throughout the intervention) the moment of completion of the intervention. Wrong expectations of the elder (follow-up of the accompaniment), and the management (sometimes ineffective) of the attachment of the agents, can generate a sense of abandonment on both sides at the end of the intervention.

Despite the presence of some constraints influencing the effectiveness and sustainability of an intervention in social isolation and loneliness in noninstitutionalized older adults, the need for increased research in this area is also evident.

## Figures and Tables

**Table 1:** Characteristics of informants.

Final Population	Volunteers from Social Organizations (n = 9) Nursing Students (n = 11) Health Personnel (n = 2)
**Average Age**	
Volunteers from Social Organizations. Nursing students. Health personnel.	53 years old (n = 8) 21 years old (n = 11) 43 years old (n = 2)
**Gender**	
Volunteers from Social Organizations. Nursing students. Health personnel.	Women (n = 5)/Men (n = 3) Women (n = 8)/Men (n = 1) Women (n = 1)/Men (n = 1)
**Average length of professional experience/volunteer activity**	
Volunteers from Social Organizations. Nursing students. Health personnel.	7.3 years. 23 years.

## Data Availability

Data are available upon reasonable request.
